# Moderate Maternal Alcohol Exposure on Gestational Day 12 Impacts Anxiety-Like Behavior in Offspring

**DOI:** 10.3389/fnbeh.2017.00183

**Published:** 2017-09-29

**Authors:** Siara K. Rouzer, Jesse M. Cole, Julia M. Johnson, Elena I. Varlinskaya, Marvin R. Diaz

**Affiliations:** Department of Psychology, Center for Development and Behavioral Neuroscience, Developmental Exposure Alcohol Research Center, Binghamton University, Binghamton, NY, United States

**Keywords:** prenatal alcohol exposure, moderate, fetal ethanol exposure, anxiety, adolescents, adult offspring

## Abstract

Among the numerous consequences of prenatal alcohol exposure (PAE) is an increase in anxiety-like behavior that can prove debilitating to daily functioning. A significant body of literature has linked gestational day 12 (G12) heavy ethanol exposure with social anxiety, evident in adolescent males and females. However, the association between non-social anxiety-like behavior and moderate alcohol exposure, a more common pattern of drinking in pregnant women, is yet unidentified. To model moderate PAE (mPAE), we exposed pregnant Sprague-Dawley rats to either room air or vaporized ethanol for 6 h on G12. Adolescent offspring were then tested on postnatal days (P) 41–47 in one of the following four anxiety assays: novelty-induced hypophagia (NIH), elevated plus maze (EPM), light-dark box (LDB) and open-field (OF). Our findings revealed significant increases in measures of anxiety-like behavior in male PAE offspring in the NIH, LDB and OF, with no differences observed in females on any test. Additionally, male offspring who demonstrated heightened anxiety-like behavior as adolescents demonstrated decreased anxiety-like behavior in adulthood, as measured by a marble-burying test (MBT), while females continued to be unaffected in adulthood. These results suggest that mPAE leads to dynamic changes in anxiety-like behavior exclusively in male offspring.

## Introduction

Maternal alcohol consumption can contribute to a variety of deficits which can be broadly defined as Fetal Alcohol Spectrum Disorders (FASDs). In-school assessments of FASDs in the United States place conservative estimates of prevalence at 1% of the population, with these numbers doubling in Italy and sextupling in South Africa (May et al., 2009). However, the Institute of Medicine’s diagnostic criteria suggests this rate may be closer to 2%–5% in the US and Europe, a likely under-estimated rate due to the stigma associated with maternal ethanol consumption (Corrigan et al., 2017). FASDs can manifest through physical, behavioral, cognitive and psychological impairments that range in classification from mild to severe (Autti-Rämö and Granström, 1991; Hamilton et al., 2003; Roebuck-Spencer et al., 2004; Kodituwakku, 2007; Simmons et al., 2010; Riley et al., 2011; Nguyen et al., 2013; Williams et al., 2014). Physical abnormalities include stunted growth and facial dysmorphia, while cognitive tests have found that children diagnosed with FASD show below-average IQs, and poor performance in complex language and arithmetic tests. Behavioral and psychological impairments include an inability to adapt socially, hyperactivity, poor focus and attention, and numerous mental health issues, including depression and anxiety. Strikingly, a considerable 21% of children diagnosed with an FASD meet the criteria for anxiety disorders (O’Connor and Paley, 2009), compared with the median prevalence rate of 8% in normal youth populations (Merikangas et al., 2009).

In humans, the severity of prenatal alcohol exposure (PAE)-induced deficits are associated with the timing and amount of alcohol exposure during gestation (O’Leary et al., 2010). Similarly, studies using animal models of PAE have recapitulated this concept by demonstrating differential patterns of anxiety-like behaviors dependent on the timing of ethanol exposure during fetal development (trimester-equivalents) and/or the dosage of alcohol administered/consumed within an exposure period (Marquardt and Brigman, 2016). Although becoming increasingly more common, the number of empirical studies utilizing lower levels of ethanol exposure are far fewer than those which model heavy drinking behavior (Valenzuela et al., 2012). This is problematic, as epidemiological data suggests that moderate alcohol consumption is the predominant drinking pattern observed in pregnant women (Ethen et al., 2009). Research incorporating low to moderate levels of PAE have repeatedly reported results which mirror findings from studies of high level-PAE, including impaired glutamatergic and GABAergic plasticity in the hippocampus, altered neurogenesis in adulthood, downregulation of retinoic acid levels during embryonic development, impaired motor coordination, increased vulnerability to and preference for abused substances, and alterations to serotonergic influence on the HPA axis (see review: Valenzuela et al., 2012).

Despite the high prevalence of moderate drinking in pregnant women, and the substantial proportion of the FASD population that meets the criteria for an anxiety disorder, studies examining the impact of moderate PAE (mPAE) on the development of anxiety are unclear. Animal models of heavy PAE demonstrate inconsistent effects on anxiety-like behavior (Osborn et al., 1998a,b; Carneiro et al., 2005; Kleiber et al., 2011; He, 2014; Liang et al., 2014), with some studies demonstrating sex-dependent changes in anxiety-like behavior in PAE offspring (Hofmann et al., 2002, 2005; Hellemans et al., 2010a; Mooney and Varlinskaya, 2011; Diaz et al., 2016). Interestingly, recent studies have identified gestational day (G) 12 as a stage in fetal development vulnerable to high-level ethanol-induced social anxiety-like alterations, regardless of rat strain (Mooney and Varlinskaya, 2011; Middleton et al., 2012; Diaz et al., 2016). Importantly, G12 corresponds with an epoch during which the amygdala, a brain region associated with the expression of anxiety, is undergoing significant development (Soma et al., 2009; Charil et al., 2010).

Based on these observations, the objective of the present study was to assess the impact of G12 mPAE on anxiety-like behavior in exposed offspring. The appearance of anxiety disorders is often observable in humans by adolescence in the general population (Beesdo et al., 2009), in individuals with FASD (O’Connor and Paley, 2009), and similarly in rats on postnatal (P)42 following G12 heavy PAE (Mooney and Varlinskaya, 2011; Diaz et al., 2016). Therefore, we focused our assessment for anxiety-like behaviors during an equivalent period of mid-to-late adolescence, in addition to one behavioral measure in adulthood.

## Materials and Methods

### Animals

Male and female adult Sprague-Dawley rats (P60+) were obtained from Envigo/Harlan (Indianapolis, IN, USA) to be bred in-house. With the exception of pregnant dams and male breeders, who were singly housed, all animals were group-housed. Animals were housed in a temperature-controlled (22°C) vivarium, and maintained on a 12:12 h light:dark cycle (lights on at 07:00 h) with *ad libitum* access to food (Purina rat chow) and water. All animal procedures were approved by the Binghamton University Institutional Animal Care and Use Committee.

### Breeding

All animals were group-housed by sex until breeding began, at which point two viable nulliparous female rats were placed in a cage with a single male breeder, and females were checked daily for pregnancy for up to four total days. Vaginal smears were collected every morning, with the first day of detectable sperm designated as G1 (Mooney and Varlinskaya, 2011). Once pregnancy was confirmed, females were single-housed and continued to receive food and water *ad libitum*. Females were weighed on G1, G10 and G20. After giving birth, dams were left with their pups for 2 days, at which time pups were counted, weighed and culled to a ratio of 5:5 males and females. Pups were then returned to their mother until weaning at P23, at which point they were separated by sex and group-housed with littermates until further testing. Additional pup weights were collected at P7 and P12; otherwise, cages were undisturbed until weaning.

### Vapor Ethanol Exposure

On G12, pregnant dams in their home cages were transferred to vapor inhalation chambers as previously described (Morton et al., 2014). Depending upon the experimental group, dams were exposed to either room air (control group) or vaporized ethanol (mPAE group) for 6 h (09:00–15:00). Dams had usual access to food and water throughout the exposure. Dams were not removed from their cages or handled at any time during this procedure. At the end of the exposure, cages were removed from vapor chambers and returned to the colony room. Food was replaced immediately after the exposure in the ethanol-exposed cages to avoid additional exposure to ethanol absorbed by the food.

In order to avoid additional stress on experimental animals, a different subset of pregnant dams was used for determination of blood ethanol concentrations (BECs), with tail-bloods collected every 2 h over a 12-h period. Dams were euthanized following the last blood collection time point.

### Behavior Testing

Beginning on P35, each animal was handled daily until the behavioral testing began, in order to reduce handling stress on day of testing. A battery of behavioral tests was performed from P41–P47, with one male and one female from a given litter randomly assigned to one of the following tests to avoid litter effects. No animal performed more than one behavioral test to avoid carryover effects from repeated testing (Doremus-Fitzwater et al., 2009). Since the experimenter was not present in the room during testing, all tests were video recorded for later scoring by an experimenter blind to the assigned conditions of each experimental animal.

### Novelty-Induced Hypophagia (NIH)

Novelty-induced hypophagia (NIH) is a validated test for anxiety-like behavior induced by changes in environmental contexts (Hunsberger and Duman, 2007; Dulawa, 2009). Rats underwent NIH testing from P41–P47, with the training period occurring from P41–P46, and a novelty-testing day occurring on P47.

#### Training Phase

On P41, rats were removed from their home-cages and isolated in a new standard cage (“training cage”) for 1 h, lined with fresh bedding and access to food and water, to acclimate to the novel environment (animals were not food or water restricted at any point during NIH testing). After the 1-h isolation period, a peanut butter cracker was placed in the cage. The animal’s interactions with the cracker were video-recorded for 15 min. After 15 min, rats were returned to their home-cages. Peanut butter crackers were weighed before and after the experiment to determine how much of the cracker had been consumed by the rat (in grams). This process was repeated for the next 5 days using the same “training cage” introduced on the first day.

#### Novelty Test

On the seventh day, rats were allowed to acclimate to their “training cage” for 1 h, as previously done within the training phase. Following this acclimation period, the animal was transferred to a novel plastic tub (58.42 cm (L) × 43.18 cm (H) × 31.75 cm (W) with no bedding/water/food) and a peanut butter cracker was immediately placed in the novel tub. The animal’s interactions with the cracker in the novel environment were video-recorded for 15 min. After 15 min, rats were returned to their home-cages. Peanut butter crackers were weighed before and after experimentation to determine consumption quantities, as the amount of cracker consumed has also previously been reported as a measure of anxiety-like behavior (Dulawa et al., 2004; Bechtholt et al., 2007; Ueda et al., 2017).

Experimental videos were later analyzed for the time taken by the animal to approach and begin consuming the cracker (latency), as well as the amount of cracker consumed.

### Elevated Plus-Maze (EPM)

In a different subset of animals, the EPM was performed on P41 as a measure of anxiety-like behavior (Hunsberger and Duman, 2007; Walf and Frye, 2007). The EPM apparatus was elevated 50.0 cm above the floor and consisted of two open and two closed arms, with each arm running 48.3 cm in length and 12.7 cm wide. Plastic edges (1.3 cm high) ran along each side of the open arms to reduce the likelihood an animal would fall off the edge. Walls surrounding the closed arms measured 29.2 cm tall. Illumination at the entrance into the open arms was set to ~90 lux. On testing day, animals were socially isolated in an unfamiliar holding cage for 1 h in a novel room in order to increase exploration in the open arms, as previous literature has demonstrated (Pellow et al., 1985; Doremus-Fitzwater et al., 2009). At the beginning of testing, each subject was placed at the center of the crossing arms, facing either open arm and permitted to freely move within the maze for 5 min. Immediately following placement of the rat in the maze, the experimenter left the room for the length of the trial. Between animals, the apparatus was cleaned with 3% hydrogen peroxide and thoroughly dried. Measures analyzed included: % of time spent in the open and closed arms, entries into the open and closed arms, as well as head dips and stretch attend postures. An animal was qualified as entering an arm when all four paws were placed in that arm.

### Light-Dark Box (LDB)

In another subset of P42 animals, the light-dark box (LDB) was performed to assess anxiety-like behaviors (Shimada et al., 1995; Bourin and Hascoët, 2003). The apparatus consisted of two adjoined chambers (34 × 24 × 24 cm), with a circular aperture (8 cm diameter) between the two chambers permitting voluntary movement between the chambers. Chambers were divided by appearance, with white and black opaque Plexiglas. The white section was deemed “the light box” and the black section was deemed “the dark box”. A black, non-translucent lid covered the dark box to prevent ambient room light from streaming in. The light box was covered by a transparent plastic lid, which allowed room light into the apparatus. Prior to testing, and between trials, the boxes were cleaned with 3% hydrogen peroxide and dried thoroughly. All testing was conducted under ambient room lighting. To begin testing, an animal was placed in the light box, facing away from the aperture between the chambers. At this time, the researcher left the room, and the animal was permitted to investigate the chambers for 5 min. This 5-min period was video recorded, and later assessed for the following measurements: time spent in each chamber, latency to enter the dark side for the first time (egress), latency to return to the light side for the first time (re-entry) and the number of transitions between chambers.

### Open-Field Test (OF)

In a different subset of P43 animals, anxiety-like behaviors were assessed using the open-field (OF; Prut and Belzung, 2003; Hunsberger and Duman, 2007). The apparatus consisted of a square box with a base (46-cm diameter) and walls (32-cm) made of black plastic. Inside the box, a center square (25-cm diameter) was outlined in red tape, providing a 10.5-cm wide space outside the center square, adjacent to the walls. The apparatus was placed on the floor and lit by ambient room lighting. Before and between trials, the box was cleaned with 3% hydrogen peroxide and dried off. To begin the trial, the subject was placed in the center square of the OF and permitted to freely roam throughout the box for 5 min. Video recordings were later scored for time spent in the center square, time spent moving throughout the box (locomotor time) and the number of entrances into the center compartment.

### Marble-Burying Test (MBT)

This test was performed on adult rats, with ages ranging from P80–90, as a means of assessing whether demonstration of anxiety-like behavior persisted into adulthood. Rats who underwent the marble-burying test (MBT) were the same rats tested in the LDB or OF as adolescents. MBT was conducted according to established protocols (Deacon, 2006), where 15 smooth marbles were placed in a small cage lined with 5 cm of bedding. Marbles were placed in a 3 × 5 grid design, with 4–5 cm spacing between each marble. A subject was placed in this cage and video-recorded for 15 min, during which time the experimenter was absent from the room. Videos were later scored by counting, in 5-min increments, the number of marbles fully buried.

### Statistics

Statistical analyses was performed using GraphPad 6 Software (Prism). Based on previous reports of G12 high-dose ethanol exposure affecting social anxiety-like behavior differently in adolescent males and females (Diaz et al., 2016), our *a priori* hypothesis was that mPAE would also differentially alter anxiety-like behavior in males and females. Therefore, we tested for sex differences, but also analyzed data from males and females separately. All measures of the EPM, LDB and OF tests were analyzed using 2 (exposure: air, ethanol) × 2 (sex: male, female) analysis of variance (ANOVA). NIH data for training days was analyzed using 2 (exposure) × 2 (sex) × 2 (day) ANOVAs, with day treated as a repeated measure. When comparing the last day of training and novelty day for NIH, latency to consume the cracker and quantity of cracker consumed were analyzed in a 2 × 2 ANOVA. In the event of significant main effects or interactions, *post hoc* Fishers LSD tests were used. Marble burying data was analyzed using a 2 (exposure) × 2 (sex) ANOVA. Significance was defined as *p* ≤ 0.05. All data are presented as mean ± standard error of the mean (SEM). A chi-square goodness of fit test determined that the distribution of sexes within behavioral testing was not significantly different ($X_{\left(3\right)}^{2}$ = 11.76, *p* < 0.01).

## Results

### Blood Ethanol Concentration

**Figure 1:**
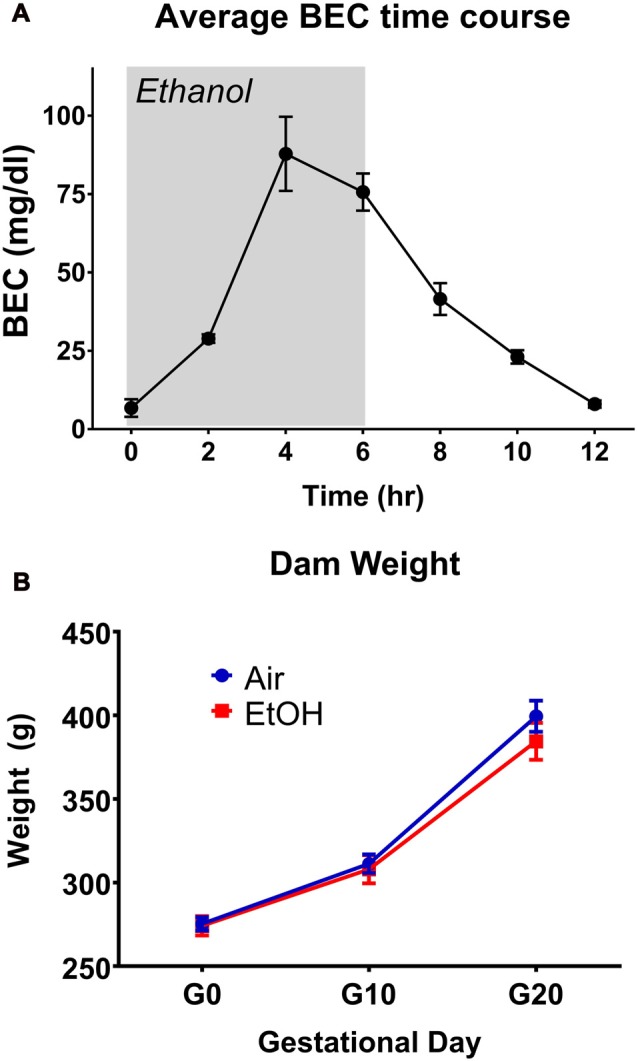
Blood ethanol concentration (BEC) time-course and dam weights. **(A)** Time-course of BECs over a 12 h period during gestational day 12 (G12) vapor ethanol exposure. Gray shade represents period of vapor ethanol exposure. **(B)** Dam weights on G0, G10 and G20.

BECs were determined at 2-h intervals for a period of 12 h, with exposure beginning at the 0-h mark and ending at 6 h (Figure 1A). This exposure resulted in a peak BEC level of 87.8 ± 11.8 mg/dL (*n* = 3), which occurred at the 4-h mark of exposure. These levels are consistent with previous literature determinations of “moderate” PAE (Valenzuela et al., 2012). Following termination of vapor ethanol exposure, BECs dropped to baseline levels within 6 h.

### G12 mPAE Paradigm Characterization

Several parameters were compared between exposure conditions (control/air and mPAE/ethanol), including dam weight on G1, G10 and G20 (Figure 1B), number of pups per litter, sex ratio per litter, length of gestation and pup weight on P2, P7 and P12 (Table 1). No significant differences were found between the exposure conditions at any measure.

**Table 1:** Means of G12 moderate prenatal alcohol exposure (mPAE) characteristics ± standard error of the mean.

Exposure	# Pups/litter	Sex ratio (% males/litter)	Gestational period (Days)	P2 Pup weight (grams)	P7 Pup weight (grams)	P12 Pup weight (grams)
Air (*n* = 19)	14.21 (± 0.61)	48.53 (± 2.73)	23 (± 0.08)	7.92 (± 0.26)	18.12 (± 1.99)	31.76 (± 2.48)
Ethanol (*n* = 22)	12.55 (± 0.77)	51.38 (± 4.20)	23 (± 0.07)	8.12 (± 0.30)	17.94 (± 1.46)	32.42 (± 2.07)

*Litter characteristics, including the number of pups born to a litter, the sex distribution across litters, the length of gestation and pup weights at P2, P7 and P12, did not differ significantly as a result of moderate prenatal exposure on G12*.

### Novelty-Induced Hypophagia (NIH)

**Figure 2:**
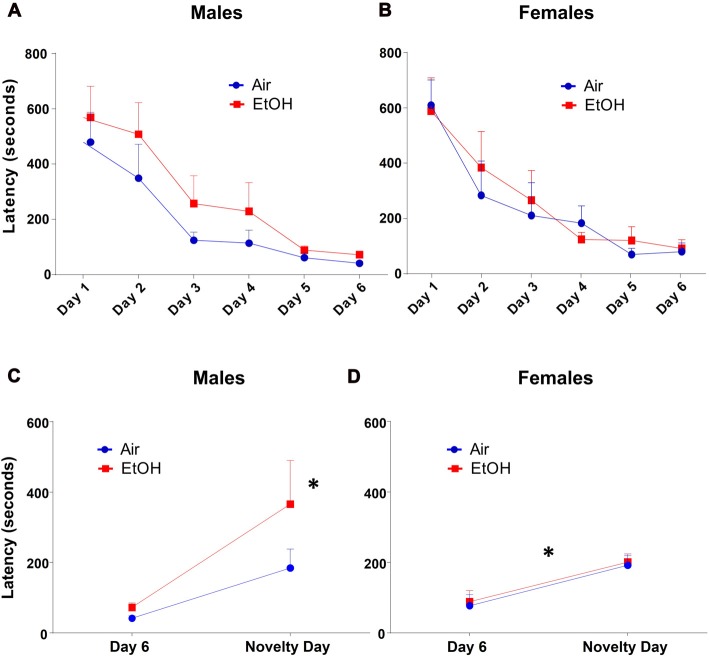
Novelty-induced hypophagia (NIH): latency to approach the cracker. Latency to approach cracker in NIH test. **(A)** Males and **(B)** females showed similar decreases in latencies to approach cracker during training days. **(C)** EtOH males demonstrated significantly increased latency on novelty day relative to day 6 of training, a difference absent in Air males; *p < 0.005 compared to EtOH males on day 6. **(D)** Air and EtOH females similarly showed increased latency on novelty day relative to day 6 of training; *p < 0.05 compared to day 6.

The NIH test began with a training/acclimation phase over six consecutive days. Latency to approach the cracker over successive training days was measured during the 15-min testing period. Both air and ethanol exposed offspring demonstrated a similar and significant decrease in latency across training days by repeated measures ANOVA (Figures 2A,B: *F*_(5,135)_ = 26.21, *p* < 0.0001). However, latency to approach the cracker on training days did not differ as a function of exposure in either males (Figure 2A: *F*_(1,28)_ = 2.445, *p* = 0.129) or females (Figure 2B: *F*_(1,26)_ = 0.127, *p* = 0.725).

To measure anxiety-like behavior induced by a novel environment, we compared the latency to consume a peanut butter cracker on novelty day to that on the previous and last acclimation day (Day 6). Using a 2 (exposure) × 2 (testing day) ANOVA (Figure 2C), we found that there was no significant effect of exposure (*F*_(1,28)_ = 2.445, *p* = 0.129) or interaction (*F*_(1,28)_ = 1.237, *p* = 0.276). However, there was a significant effect of testing day (*F*_(1,28)_ = 10.34, *p* = 0.003). Fisher’s LSD tests revealed a significant delay in approach to the cracker in mPAE males from Day 6 to novelty day (*n* = 7, *p* < 0.005), where no significant delay was seen in control males (*n* = 8, *p* = 0.148).

Similar to males, a 2 (exposure) × 2 (testing day) ANOVA from latencies in females showed that there was no significant effect of exposure (*F*_(1,26)_ = 0.127, *p* = 0.725) or an interaction (*F*_(1,26)_ = 0.002, *p* = 0.966). There was a significant effect of testing day for latency (*F*_(1,26)_ = 15.86, *p* = 0.0005); however, both groups were found to similarly increase from Day 6 to novelty day (Figure 2D). Fisher’s LSD tests revealed significant increases in approach latency between control females from Day 6 to novelty day (*n* = 8, *p* = 0.011) as well as in mPAE females (*n* = 8, *p* = 0.008).

**Figure 3:**
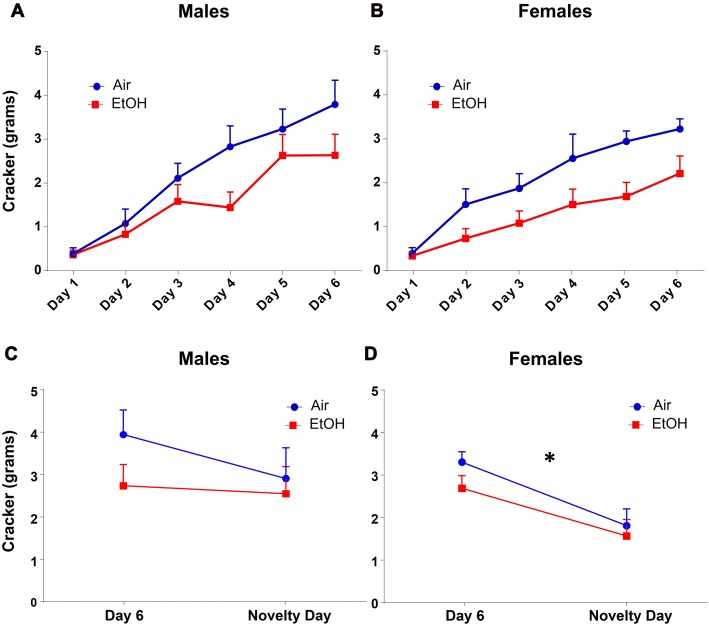
NIH: cracker consumption. Quantity of cracker consumption during NIH test. **(A)** Males and **(B)** females both increased amount of cracker consumption over training days, regardless of prenatal exposure. Quantity of cracker consumed on novelty day compared to day 6 of training in **(C)** males was not different in either exposure group. **(D)** Females from both exposures showed significantly decreased cracker consumption on novelty day relative to day 6, *p < 0.05 compared to day 6.

Since amount of food eaten can also indicate changes in anxiety-like behavior, we also analyzed amount of cracker consumption during this test. We found that quantity of cracker eaten significantly increased across training days similarly in both males and females using a repeated measures ANOVA, regardless of prenatal exposure (Figures 3A,B: *F*_(5,135)_ = 63.65, *p* < 0.0001). There was also a main effect of exposure on cracker consumption on training days with EtOH animals consuming significantly less than air animals (*F*_(1,27)_ = 5.165, *p* = 0.031).

As with latency, based on our *a priori* hypothesis, when examining the difference in cracker consumption between the last training day and the novelty day, we found no effect of exposure (*F*_(1,28)_ = 1.613, *p* = 0.214), day (*F*_(1,28)_ = 0.988, *p* = 0.328), or interaction (*F*_(1,28)_ = 0.473, *p* = 0.497) in males (Figure 3C). In females (Figure 3D), while we found no effect of exposure (*F*_(1,26)_ = 1.585, *p* = 0.219) or an interaction (*F*_(1,26)_ = 0.297, *p* = 0.591), there was a significant effect of day (*F*_(1,26)_ = 14.71, *p* = 0.0007). Fisher LSD tests showed a significant decrease in cracker consumption from Day 6 to novelty day in both controls (*p* = 0.006) and mPAE females (*p* = 0.023).

### Elevated Plus-Maze (EPM)

Evaluation of anxiety-like responses using the EPM revealed no significant main effects of exposure (*F*_(1,32)_ = 0.053, *p* = 0.819) or sex (*F*_(1,32)_ = 2.585, *p* = 0.118), nor a significant interaction of these effects (*F*_(1,32)_ = 0.446, *p* = 0.509) for % of time spent in the open and closed arms. In regard to open arm entries, no significant effect of exposure was found (*F*_(1,32)_ = 0.015, *p* = 0.904) nor an interaction between treatment and sex (*F*_(1,32)_ = 0.730, *p* = 0.400). However, a significant effect of sex was found *F*_(1,32)_ = 5.373, *p* = 0.027), and *post hoc* comparisons revealed this effect was significant only between males and females in the ethanol group (*t* = 2.243, *n* = 9, *p* = 0.032), but not the control group (*t* = 1.035, *n* = 9, *p* = 0.308). Additionally, no significant differences were found within any other measure of this test: closed arm entries (Exposure: *F*_(1,32)_ = 0.290, *p* = 0.594; Sex: *F*_(1,32)_ = 0.185, *p* = 0.670, Interaction: *F*_(1,32)_ = 1.159, *p* = 0.290), stretch attends (Exposure: *F*_(1,27)_ = 0.274, *p* = 0.605; Sex: *F*_(1,27)_ = 0.001, *p* = 0.972, Interaction: *F*_(1,27)_ = 0.023, *p* = 0.881) and head dips (Exposure: *F*_(1,27)_ = 1.01, *p* = 0.324; Sex: *F*_(1,27)_ = 2.706, *p* = 0.112, Interaction: *F*_(1,27)_ = 1.390, *p* = 0.247; Table 2).

**Table 2:** Elevated plus-maze values across sexes: means ± standard errors and *p*-values comparing control and mPAE animals.

Mean ± SEM	Control males (*n* = 9)	mPAE males (*n* = 9)	Control females (*n* = 9)	mPAE females (*n* = 9)
% Open arm time	28.08 ± 3.16	30.08 ± 3.29	23.78 ± 6.51	19.68 ± 4.53
Open arm entries	7.11 ± 0.86	7.78 ± 0.70	5.78 ± 1.14	4.89 ± 0.89
% Closed arm time	71.92 ± 3.16	69.96 ± 3.30	76.22 ± 6.51	80.32 ± 4.53
Closed arm entries	12.44 ± 1.26	11.89 ± 0.48	11.78 ± 0.89	13.44 ± 1.28
Stretch attends	6.33 ± 0.75	5.89 ± 1.40	6.56 ± 1.04	5.75 ± 0.75
Head dips	10.56 ± 1.37	10.89 ± 1.39	9.67 ± 2.33	5.5 ± 0.96

*No measure of the elevated plus maze varied significantly across exposure groups, including % time in the open arm, % time in the closed arm, number of open arm entries, number of closed arm entries, number of stretch attends and number of head dips*.

### Light-Dark Box (LDB)

**Figure 4:**
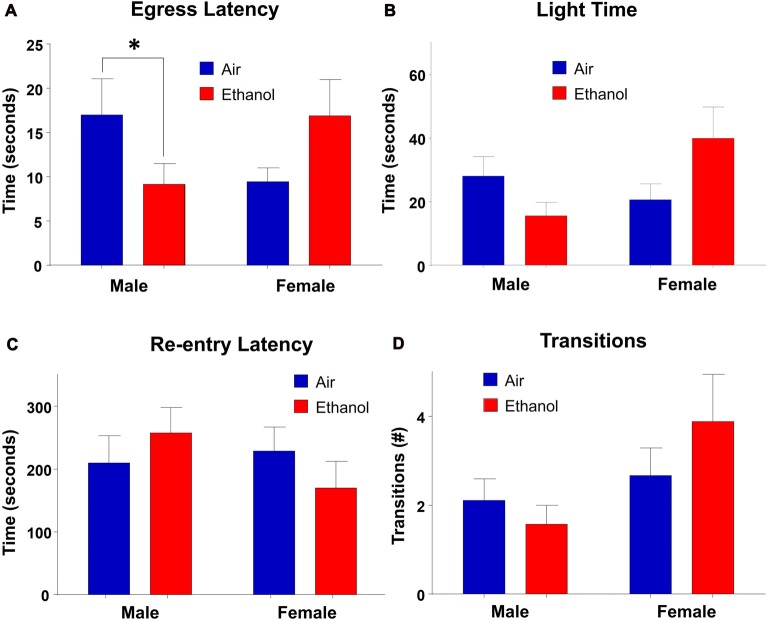
Light-dark box (LDB). Measures of anxiety-like behavior in the LDB. **(A)** Egress latency in males and females. Moderate prenatal alcohol exposure (mPAE) males demonstrated significantly shorter egress latency compared to control males, with no differences observed in females. There was no effect of exposure in either males or females in **(B)** time spent in the light side, **(C)** re-entry latency, or **(D)** number of transitions between chambers. *p = 0.05 compared to Air males.

In the LDB test, there were no main effects of exposure (*F*_(1,30)_ = 0.245, *p* = 0.624) or sex (*F*_(1,30)_ = 1.463, *p* = 0.236) on time spent in the light side of the chamber. However, there was a significant interaction between the two variables (*F*_(1,30)_ = 5.184, *p* = 0.030; Figure 4B). *Post hoc* LSD tests revealed the only comparison which differed significantly was between ethanol-exposed males and females (*n* = 7–9, *p* = 0.024). Neither males (*n* = 7–8, *p* > 0.232) nor females (*n* = 9, *p* = 0.051) differed across exposure, although there was a trend in females, with ethanol-exposed females spending more time in the light side of the chamber than air-exposed females. In measures of egress latency, there was a significant interaction (*F*_(1,30)_ = 7.019, *p* = 0.013) with no main effects of treatment (*F*_(1,30)_ = 0.110, *p* = 0.743) or sex (*F*_(1,30)_ = 0.088, *p* = 0.769; Figure 4A). *Post hoc* comparisons revealed that the only groups which varied significantly were air and ethanol-exposed males, with ethanol exposed males demonstrating significantly lower egress latencies than controls (*n* = 7–9, *p* = 0.050). Although not statistically significant, there was a trend toward a difference between ethanol-exposed males and females at *p* = 0.0527 (*n* = 7–9). In regard to re-entry latency, no significant effects of exposure (*F*_(1,30)_ = 0.017, *p* = 0.897) or sex (*F*_(1,30)_ = 0.679, *p* = 0.417) were found, nor an interaction between the two variables (*F*_(1,30)_ = 1.638, *p* = 0.210; Figure 4C). Similarly, there was no significant effect of exposure (*F*_(1,30)_ = 0.220, *p* = 0.643), sex (*F*_(1,30)_ = 3.895, *p* = 0.058) or an interaction of the independent variables (*F*_(1,30)_ = 1.465, *p* = 0.236) in transitions (Figure 4D).

### Open Field (OF)

**Figure 5:**
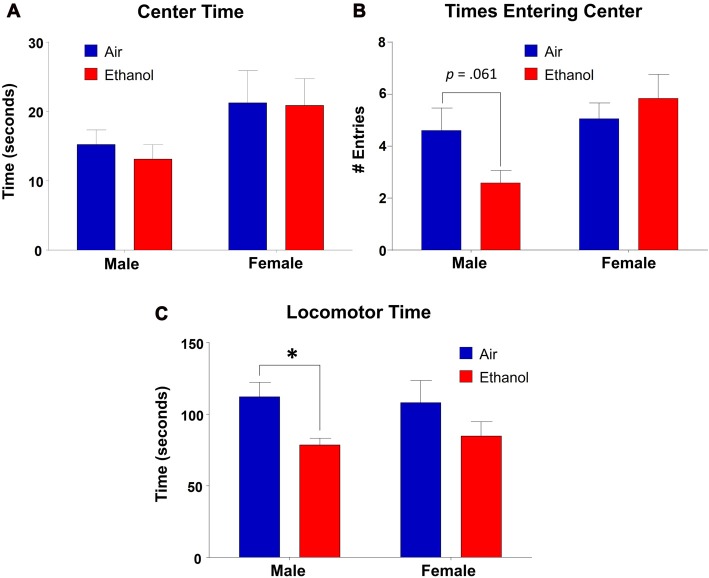
Open-field (OF) test. Anxiety-like behavior measures in the OF apparatus. **(A)** Time spent in the center did not vary significantly by exposure in either males or females. **(B)** Center entries in mPAE males was lower than controls, but did not reach significance. This effect was not significant in females. **(C)** mPAE males spent significantly less time moving than control males, but this was not observed in females. *p < 0.05 compared to Air males.

We found that time spent in the center of the OF did not differ as a function of exposure (*F*_(1,31)_ = 0.143, *p* = 0.708), but there was a significant effect of sex (*F*_(1,31)_ = 4.453, *p* = 0.043), with no interaction between the variables (*F*_(1,31)_ = 0.072, *p* = 0.791; Figure 5A). However, *post hoc* LSD revealed no significant differences in comparisons across exposures in either males (*n* = 9, *p* = 0.646) or females (*n* = 8–9, *p* = 0.939). In center entries, there was a significant sex effect (*F*_(1,31)_ = 6.14, *p* = 0.019), with no effect of exposure (*F*_(1,31)_ = 0.683, *p* = 0.415) or an interaction (*F*_(1,31)_ = 3.526, *p* = 0.070; Figure 5B). While *post hoc* tests revealed no significant differences across exposure in females (*n* = 8–9, *p* = 0.469), ethanol-exposed males demonstrated fewer entrances into the center than controls, although this did not reach significance (*n* = 9, *p* = 0.061). Additionally, ethanol-exposed animals did differ significantly as a function of sex (*n* = 9, *p* = 0.003). As a separate measure, we assessed locomotor activity by analyzing the amount of time spent moving throughout the OF apparatus (including both time moving in the periphery and in the center). Surprisingly, we found a significant effect of exposure in this measure (*F*_(1,31)_ = 7.497, *p* = 0.010), with no effect of sex (*F*_(1,31)_ = 0.010, *p* = 0.919) or an interaction (*F*_(1,31)_ = 0.246, *p* = 0.624). *Post hoc* tests revealed that ethanol-exposed males spent significantly less time moving throughout the OF than control males (*n* = 9, *p* = 0.027), a difference absent in females across exposure (*n* = 8–9, *p* = 0.128; Figure 5C).

### Marble Burying Test (MBT)

**Figure 6:**
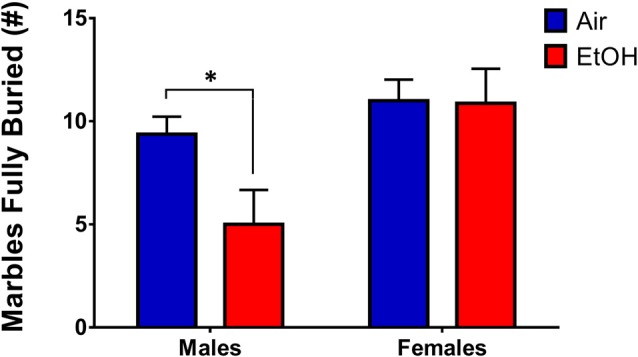
Marble-burying test (MBT). Marble-burying in adult rats who previously underwent LDB and OF tests. mPAE males buried significantly less marbles than controls after 15 min, whereas females did not demonstrate a difference in number of marbles fully-buried; *p < 0.05 compared to Air males.

To assess persistent changes in anxiety-like behavior into adulthood, we tested offspring who had previously undergone LDB or OF tests in in the MBT. Since we utilized animals that had undergone LDB and OF testing for the MBT, in order to continue using a litter as the unit of determination (*n* = 1), we averaged data from same-sex siblings. A 2 (sex) × 2 (exposure) ANOVA revealed a significant sex effect (*F*_(1,12)_ = 7.638, *p* = 0.017), with no significant effect of exposure (*F*_(1,12)_ = 2.750, *p* = 0.123) or an interaction (*F*_(1,12)_ = 2.453, *p* = 0.143). However, Fishers LSD test found a significant difference in # of marbles buried in males (*p* = 0.041), indicating that mPAE males buried significantly fewer marbles than controls (Figure 6). There was no difference in females (*p* = 0.949).

## Discussion

Our study aimed to investigate the influence of G12 mPAE on expression of anxiety-like behaviors in adolescent offspring. Under a model of mPAE utilizing vaporized ethanol exposure on G12, in which BECs gradually increased, male adolescent offspring demonstrated increased anxiety-like behavior in three out of four behavioral assays, whereas females did not show mPAE-associated anxiety-like alterations across any test. In adulthood, mPAE male offspring demonstrated a decrease in anxiety-like responses in the MBT, a difference absent in adult females.

Pregnant Sprague-Dawley rats were exposed to either room air or vaporized ethanol for 6 h, resulting in average peak BECs of ~87 mg/dL, consistent with a model of moderate exposure (Valenzuela et al., 2012). One important factor of using this route of administration is that the rise in BECs is gradual, indicated by the peak BECs following 4 h of vapor ethanol exposure. This is representative of individuals who pace themselves while drinking alcohol. Importantly, this gradual rise in BECs is unattainable through a single instance of intraperitoneal injection, intragastric administration of ethanol, voluntary ethanol drinking, or forced liquid ethanol diet. Unlike high-level ethanol exposure studies, which have found underdevelopment in ethanol-exposed pups (Dursun et al., 2006; Brocardo et al., 2012; Ávila et al., 2016; Huebner et al., 2016; Liu et al., 2016), G12 mPAE did not produce significant phenotypic differences in litter characteristics and pup weights. This is consistent with previous studies of acute heavy PAE on G12, which also found no effect of exposure on litter characteristics (Diaz et al., 2016). However, it is worth noting that alterations in litter characteristics and pup weight are suggested to be dose- and exposure time-dependent (Abel, 1996; Marquardt and Brigman, 2016). Additionally, although it is unknown whether this vapor exposure alters caloric intake in the pregnant dams, dam weight gain was not significantly affected by the exposure at the end of gestation (G20).

Another important consideration regarding our mPAE model is the presence of sex differences. Many studies have shown that PAE can result in sex-related impairments, but many variables must be considered, including dose of ethanol, timing of exposure, age of testing and the measured outcome. Specifically, with ethanol exposure on G12, sex-specific anxiogenic social deficits have been reported in offspring following high level-exposure (Diaz et al., 2016), with both sexes demonstrating significantly reduced social behaviors as a result of PAE in Sprague-Dawley rats. However, this effect differed between sexes depending upon the age at which subjects were tested, with males showing increased social anxiety as early-adolescents, while females exhibiting social anxiety as late-adolescents and adults. In contrast, although Mooney and Varlinskaya (2011) found increases in social anxiety-like alterations following G12 exposure in both sexes in Long Evans rats, these alterations were more pronounced in males than females. Interestingly, only females showed social anxiety-like alterations as adults when exposure occurred on G15 (Varlinskaya and Mooney, 2014), whereas both adolescent males and females showed decreased levels of social anxiety relative to controls, indexed via increased social investigation, when ethanol exposure occurred on G7 (Mooney and Varlinskaya, 2011).

In the present study, however, we found that anxiogenic effects of G12 mPAE were predominantly expressed in male offspring when tested as mid-to-late adolescents. This suggests that females may be more resilient to the effects of ethanol exposure during the beginning of the 2nd trimester-equivalent, although this resiliency may be overcome by higher doses of ethanol (Diaz et al., 2016). Interestingly, although not anticipated, under three different behavioral tests, we observed a sex difference in ethanol-exposed animals that was not present in air-exposed groups. Although direct comparisons to control animals within sex did not reach significance, egress latency and light time in the LDB, as well as center entries in the OF revealed a pattern of behavior in which males showed increased anxiety-like behavior following mPAE, and mPAE females demonstrate the opposite effect, inclining toward reduced anxiety-like behavior. This significant difference between ethanol males and females was further seen in open arm entries of the EPM. Together, this data appends research suggestive of differential effects of alcohol on sexes, and highlights the importance of looking at both sexes in research involving prenatal exposure to ethanol.

Males demonstrated increased anxiety-like behavior on the NIH test following mPAE. Specifically, when comparing latency to approach cracker on the last day of training and the novelty testing day, mPAE males exhibited a significantly longer delay in approaching the cracker in a novel environment relative to the final training day, and this was not observed in control animals. In female offspring, both control and mPAE groups showed significant increases in latency to approach the cracker. These findings suggest that in mPAE males, the unfamiliar environment is anxiety-provoking, even after becoming familiarized to the stimulus. Conversely, in females, exposure to a novel environment equally induces anxiety regardless of prenatal treatment. Based on these findings, it is possible that there was a ceiling effect in females, however, this will have to be further assessed in the future. Nevertheless, these data suggest that anxiety-like behavior across sexes may vary depending upon the task/event that induces anxiety.

Behavioral deficits in mPAE males were also observed in the LDB through egress latency (the first transition from the light to dark compartment), but in no other LDB measure. These data suggest that in the presence of an aversive, high-lit environment, mPAE males were much quicker to leave the anxiety-inducing environment than controls. Surprisingly we did not find a difference in time spent in the light, which is the more typically reported measure of anxiety-like behavior in the LDB. It is possible that our test was too anxiogenic as control animals spent only ~10% of the time in the light side, which is significantly lower than control animals seen in other studies utilizing the LDB as a measure of anxiety-like behavior (Slawecki, 2005; Kupferschmidt et al., 2010; Arrant et al., 2013; Lee et al., 2016). Nevertheless, although not significant, mPAE males did show a reduction in light time. One potential reason for why this effect was not more dramatic is because animals did not undergo a habituation period for this test as compared to the training phase of the NIH. However, animals were handled for a week prior to testing to avoid additional handling stress that could have influenced behavior in this test. Interestingly, in another subset of animals that were unhandled prior to LDB testing, we found much higher variability in all measures (data not shown), suggesting that handling profoundly influences behavior in this task. Regardless, the difference in egress latency in mPAE males was significant, consistent with the observed anxiety-like behavior seen in the NIH.

Increased anxiety-like behavior was evident in mPAE males when tested in the OF. The OF is an established test of anxiety-like behavior that relies on the assumption that anxious animals spend significantly less time in the center square and more time near the walls in the outer compartment (Carter and Shieh, 2015). Furthermore, anxious animals demonstrate less exploratory behavior, including transitioning between and moving throughout compartments. Although we observed no differences in time spent in the center across groups, mPAE males showed reduced center entries relative to controls (although this did not reach statistical significance). One factor that must be considered with this OF test is locomotor behavior, which has been shown to be altered by PAE (Brys et al., 2014; Skorput et al., 2015; Muñoz-Villegas et al., 2017). Analysis of the time each group spent moving throughout the OF apparatus (locomotor time) revealed a significant decrease in locomotor activity in mPAE males relative to control males. This effect was not seen nor expected in females, as prenatal exposure did not produce differences in either center-time or center entries for females. While these results are consistent with previous observations associating hypolocomotion with an anxiogenic phenotype (Bagdy et al., 2001), it is difficult to fully attribute the reduced center entries to hypolocomotion, as we did not examine distance moved. Additional assessment should include distance moved to determine the extent of the observed hypolocomotor activity. Interestingly, although most studies indicate that PAE increases locomotor activity (Brys et al., 2014; Skorput et al., 2015; Muñoz-Villegas et al., 2017), there is research that has demonstrated decreased locomotor activity following PAE as well (Elibol-Can et al., 2014). Future studies should better examine the influence of G12 mPAE on locomotor activity and the relationship between locomotor activity and anxiety-like behaviors, particularly in the context of PAE.

In contrast to the previous tests of anxiety-like behavior, we found no differences in anxiety-like behavior across exposure conditions in the EPM. Previous research of heavy PAE has produced conflicting results within this test: one study in adolescent mice failed to find an anxiety-altering effect of high level-ethanol exposure throughout gestation on EPM performance (Boehm et al., 2008). In a study utilizing Sprague-Dawley rats, an effect of prenatal exposure was only observed in the presence of PAE combined with chronic mild stress, but not in PAE alone (Hellemans et al., 2010b). Two studies which assessed prolonged mPAE throughout gestation uncovered contradictory results in the EPM: an anxiogenic effect in Sprague-Dawley rats (Cullen et al., 2013) and no effect in offspring of Wistar rats (Barbaccia et al., 2007). Furthermore, when ethanol exposure occurred during the 3rd trimester-equivalent, anxiety-like behavior of adolescents in the EPM was only observed following brief high level-exposure (P3–5; Baculis et al., 2015), but not prolonged and moderate exposure (P2–12; Diaz et al., 2014). Together, these findings imply that the efficiency of the EPM as a measure of PAE-induced anxiety-like behavior is complex. Another interpretation of our results, whereupon the expression of anxiogenic behavior appears in only three of four behavioral assays, is that mPAE-induced anxiety may express itself in a context-dependent manner. It is worth mentioning that the limits of the EPM as a measure of anxiety-like behavior may be attributable to the form of anxiety it empirically measures: a situation-dependent anxiety/fear, as opposed to generalized anxiety behaviors (Falter et al., 1992). Therefore, our findings may suggest that G12 mPAE is somewhat specific in the forms of anxiety it can induce.

In addition to the effects observed in adolescent animals, we were also interested in determining whether anxiogenic behavior persevered into adulthood, as it has been suggested that PAE also increases the likelihood for development of anxiety symptomology in adults (Steinhausen and Spohr, 1998; Hellemans et al., 2010b; He, 2014). Surprisingly, we discovered a reversal of anxiety in mPAE male animals in adulthood wherein adult males (who as adolescents demonstrated increased anxiety in the LDB and OF) now showed significantly less anxiety (burying of less marbles) than their control counterparts. This was unexpected, as previous research into the effects of PAE on generalized anxiety in adult animals has repeatedly revealed significant increases in anxiety-like behavior (Zhou et al., 2010; Cullen et al., 2013; Elibol-Can et al., 2014). However, there are some studies which have contradicted these reports, finding either a complete lack of an effect (Sanchez Vega et al., 2013) or a very mild behavioral effect (Dursun et al., 2006). Overall, there seems to be a great deal of uncertainty within this body of literature as to the long-term impact of PAE. However, we previously found that G12 high level-exposure produces similar decreases in anxiety-like behavior in the EPM of adults that show increased social anxiety-like behavior at that same age (Diaz et al., 2016). Within our own results, one potential explanation for this shift is that compensatory adaptations may be engaged from adolescence into adulthood, which over-correct for anxiety and results in an opposite, yet inappropriate anxiety-like response. It is also important to note that the MBT may not directly measure anxiety-like behaviors, as some have defined it more as a measure of obsessive-compulsive and perseverative behavior than anxiety-like behavior (Takeuchi et al., 2002; Thomas et al., 2009).

In conclusion, this study demonstrates that G12 mPAE produces anxiogenic responses in male offspring during adolescence, but not in adulthood. Surprisingly, this exposure produces no effect in female offspring at either age. Studies are warranted to identify neurobiological mechanisms associated with the age-dependent expression of anxiety-like behaviors in males and resilience in females which may be altered by exposure during this gestational period.

## Author Contributions

MRD and SKR designed research; SKR, JMC and JMJ performed research; SKR, JMJ and EIV analyzed data; SKR, EIV and MRD wrote the manuscript.

## Conflict of Interest Statement

The authors declare that the research was conducted in the absence of any commercial or financial relationships that could be construed as a potential conflict of interest.
